# Epithelial dynamics during early mouse development

**DOI:** 10.1016/j.gde.2021.11.006

**Published:** 2021-12-17

**Authors:** Shifaan Thowfeequ, Matthew Stower, Shankar Srinivas

**Affiliations:** 1 Department of Physiology, Anatomy and Genetics, South Parks Road, University of Oxford, Oxford, OX1 3QX, UK

## Abstract

The first epithelia to arise in an organism face the challenge of maintaining the integrity of the newly formed tissue, while exhibiting the behavioral flexibility required for morphogenetic processes to occur effectively. Epithelial cells integrate biochemical and biomechanical cues, both intrinsic and extrinsic, in order to bring about the molecular changes which determine their morphology, behavior and fate. In this review we highlight recent advances in our understanding of the various dynamic processes that the emergent epithelial cells undergo during the first seven days of mouse development and speculate what the future holds in understanding the mechanistic bases for these processes through integrative approaches.

## Introduction

**Figure 1:**
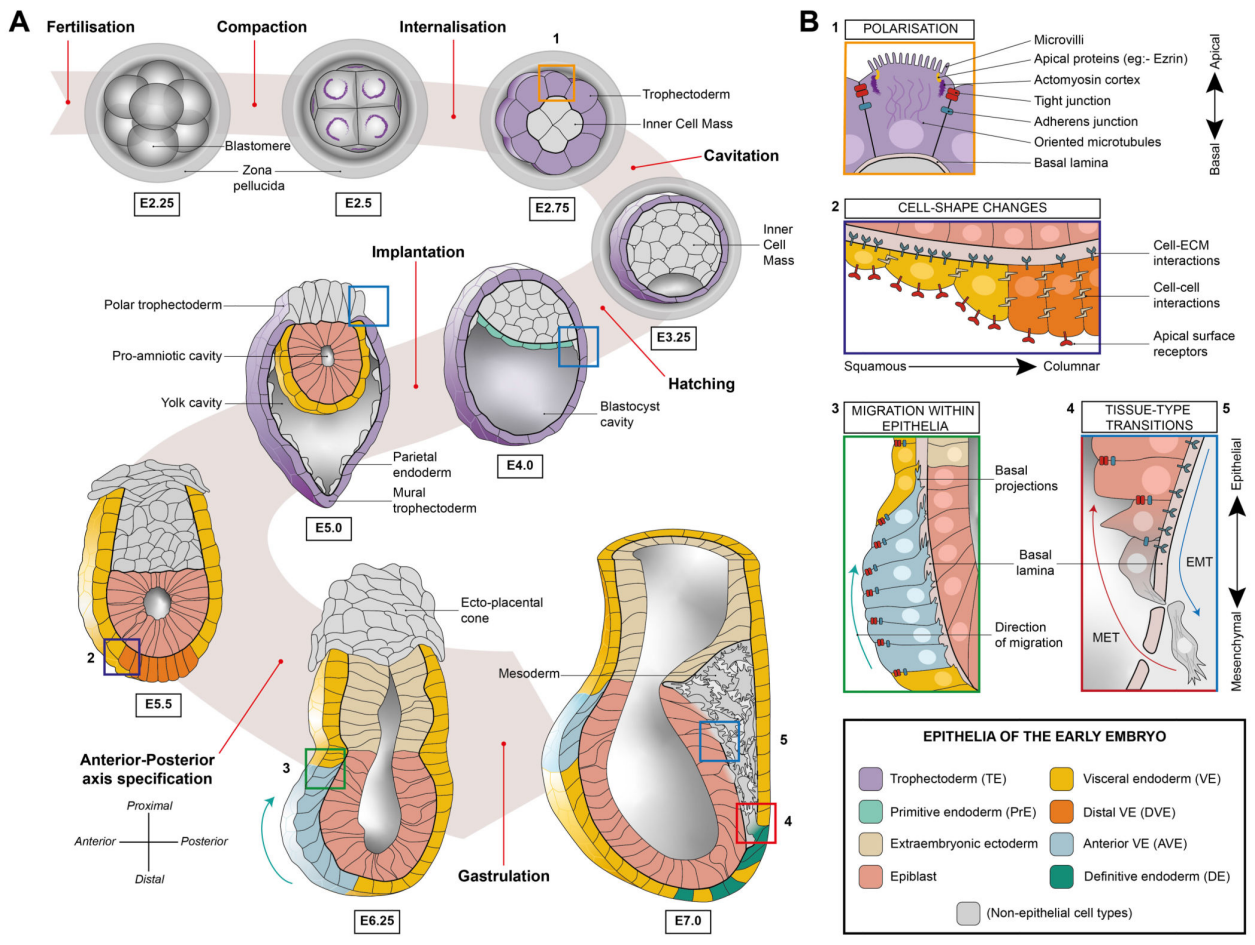
Epithelial dynamics during the first seven days of mouse development. **(A)** The various epithelia of the early mouse embryo and the sequence in which they emerge, relative to key events during the first seven days of development leading up to gastrulation. All epithelial tissues are coloured (see key) while non-epithelial tissues are shown in grey. From E5.5 onwards the TE, which encloses the whole embryo within the yolk cavity, is not shown for the sake of clarity. The hydraulic fractures between cells seen in the E3.5 blastocyst disappear as the blastocyst cavity gradually expands to occupy a significant volume of the blastocyst. Inner cell mass segregates to give rise to the epiblast and primitive endoderm, both of which subsequently epithelialise. (**B)** Different dynamic processes observed within the epithelia of the early embryo. The numbers and the colour of the box outlines correspond to regions highlighted in 1A, showing examples of where these events first occur.

Epithelia are fundamental tissue structures comprising of sheets of interconnected cells that act as physical and chemical barriers between different tissues, or tissues and their surroundings. They are highly dynamic and can undergo characteristic changes in shape and size, driven by regionalised, coordinated cell behaviours. Epithelia are critical regulators of morphogenesis at multiple levels including tissues, organs, and ultimately the shape of the whole organism. During early mouse embryogenesis, cells differentiate into embryonic and extraembryonic tissues, giving rise to some of the first epithelia of the organism (Figure 1A). The cells within these epithelia display a wide-range of behaviours including; cell polarisation, cell shape changes, tissue-type plasticity and cell migration (Figure 1B). Due to the relatively small number of cells and the organisational simplicity, the early mouse embryo allows epithelial behaviours to be studied in exquisite detail, and in isolation from major physiological influences, making this system an excellent model for understanding mammalian epithelial dynamics. Here, we give a broad overview of the cellular phenomenon leading to the origin of various epithelial tissues during the first week of development in the mouse embryo.

## Assembling epithelia

Epithelia can be seen as the default type of eumetazoan cells as they represent the first organised tissue-type to arise during development. Apicobasal polarity is a defining feature of epithelia, whereby the component cells interact with the extracellular basal lamina along their basal side, while maintaining strong interactions with each other through intercellular junctions at their apical side. Blastomeres of the late 8-cell embryo possess proto-epithelial characteristics such as the accumulation of E-cadherin at cell-cell interfaces which, along with cortical actomyosin-mediated tension, helps to drive compaction [1–5]. At this time, they also acquire the ability to sense their position within the embryo from the proportion of exposed surface area, rendered as differences in YAP localisation [6,7]. The resultant increase in cell-cell contact helps the formation of mature adherens and tight junctions and the consolidation of apicobasal polarity, with an Ezrin- and microvilli-rich apical domain facing the outside [8]. During subsequent cell-divisions, daughter cells that inherit this domain and retain less contractile ability will continue to remain polarised and on the outside [9,10], although cells dividing symmetrically or at an oblique angle can still be internalised [11,12], through mechanisms of apical constriction [13,14]. Ultimately the polarised outer cells generate the first epithelium, the trophectoderm (TE) [15], and completely envelop the non-polar highly-contractile inner cells that form the inner cell mass (ICM). Actin rings formed around a network of polar microtubules at the apical domains of TE cells expand to cell-cell junctions and undergo a tension-dependant zippering mechanism giving the epithelium greater integrity and sealing the embryo for blastocyst cavity expansion [16]. Waves of actomyosin contractility help to resolve fractures that appear in-between intercellular spaces, leading to formation of the blastocoel cavity [17,18]. Extensive crosstalk between all three cytoskeletal systems is thought to accentuate apical-basal asymmetries during these early epithelialisation events [19]. The presence of the polarised apical domain also inhibits the Hippo pathway in outer cells, permitting YAP-mediated activation of a downstream TE-specific gene expression programme [20]. The mechanical properties of these early epithelia change as the embryo grows, and advances have been made in measuring the physical forces at play within the pre-implantation embryo [14,21–23] that allows us to start integrating our relatively detailed understanding of the molecular genetics of TE specification with changes in its mechanical properties.

The ICM segregates into two epithelial tissues, the Epiblast and Primitive Endoderm (PrE) through a MAPK dependent pathway [24]. The epithelialisation and cavitation of the epiblast is contrasted from that of the TE, as cells initially polarise through extracellular matrix (ECM)-stimulated apical constrictions [25,26]. The lumen created at the centre of the epiblast expands to become the proaminiotic cavity on the apical side, while cells ‘share’ a basement membrane on their basal sides with the PrE-derived Visceral Endoderm (VE) epithelium. This *de novo* epithelialisation is associated with fate commitment decisions whereby epiblast cells downregulate their naïve pluripotent genetic profile and commit to a primed state [27]. Both the epiblast cells’ competency to epithelialise and their exit from naïve pluripotency occur in concert and have been shown to be regulated by the transcription factor Tcf15 [28]. They are accompanied, at least *in vitro*, by a disengagement of the plasma membrane from the underlying actomyosin cortex [29] and acquisition of the ability to respond to mechanical stimuli [30]. The epithelialised epiblast then forms the starting material for gastrulation in amniotes. This evolutionarily-conserved, epithelialised epiblast in amniotes (in contrast to non-amniotic vertebrates) is thought to be associated with the shift from a circumblastoporal mode of gastrulation to a posterior-epiblast restricted mechanism [31,32].

## Generating heterogeneities within epithelia

As development progresses, heterogeneities emerge within epithelia of the early embryo. Recent single-cell molecular approaches have helped to unravel some of the transcriptomic, epigenetic and proteomic changes that precede the emergence of a diversity of overt behavioural differences among epithelial cells that are, in turn, responsible for subsequent morphological transformations [33–38]. These heterogeneities might arise through an accentuation of biases in gene expression noise, to bring about deterministic fate choices [34]. They can also occur by stochastic uneven distribution, or more regulated subcellular compartmentalisation, of the transcriptome or proteome prior to and during cell division [39]. Depending on their position within an embryo, cells can also respond differently to extrinsic factors, both biochemical and biomechanical, generating intrinsic changes that determine their individual fates. The process of generating molecular and morphological diversity amongst cells is closely intertwined, making it a challenge to delineate cause from effect.

Cell-shape changes can initially result from exogenous mechanical forces generated through events such as expanding intra-embryonic cavities or changing topology of the surrounding tissue following implantation. These changes are stabilised by modifications to the cytoskeletal and adhesive architecture of cells through alterations to gene expression or the proteome [40]. The resulting molecular dynamics manifested as altered cellular properties (proliferation index, packing density, cell-shape, and cell motility) generate intracellular mechanical traction which is detected by mechanosensory pathways, read by the cytoskeletal/adhesion apparatus and affect epigenetic plasticity and gene regulatory networks to reinforce the commitment of cells to a specific differentiation trajectory [41]. It has been recently theorised that these integrative molecular changes can form the basis of a biomechanical memory epithelial cells retain, even as they undergo later transformations [42]. Cell-cycle dynamics have also been shown to regulate differentiation and cell-shape, by generating endogenous active stresses needed to remodel epithelial monolayers [43] and could be of significance to epithelia within the embryo.

A notable example of cell-shape changes occurring within an epithelium is the conversion of a subset of VE cells, the distal visceral endoderm (DVE), from a squamous to a columnar epithelium. As the mouse embryo acquires its characteristic cup-shape with the expansion of the pro-amniotic cavity, the DVE is specified at the point of maximum curvature, at the distal extreme of the egg-cylinder (Figure 1A). In addition to its unique morphological attributes, the DVE is also marked by a distinct gene expression profile which distinguishes it from the surrounding VE cells [35,44]. Whether cell-shape changes within the DVE are directly due to anisotropies of mechanical stresses or a consequence of altered molecular dynamics has not yet been elucidated. These possibilities are not mutually exclusive and both directly relate to the position of cells within the geometric constraints of the embryo. It has been suggested that a BMP signal emanating from the extraembryonic ectoderm (ExE) could play a role in specifying the DVE at the distal tip, furthest away from the source of the morphogen [45,46]. Once heterogeneities of cell shape are established, they can be leveraged to modulate the response to long-range morphogens through changes to cell-surface receptor localisation [47]. We can also postulate that alterations to signalling across shorter distances would occur as the degree of contact with neighbouring cells and the ECM change. In the case of the DVE, the cell-shape transformation is accompanied by gene expression changes [48], priming these static cells to undergo a phase-transition and become the motile anterior visceral endoderm (AVE) which specifies the anterior-posterior axis of the embryo (discussed below).

Given the crucial function of the AVE in axial patterning, the mechanism that induces the DVE would have to accommodate the inter-species variation in shapes seen between mammalian embryos. While the mouse embryo is ‘cylindrical’ in shape at this stage, the equivalent stage human embryo is ‘disk’ shaped [49]. Cells similar to the mouse DVE/AVE in terms of their columnar morphology can be seen in the hypoblast (equivalent to the mouse VE) in historical sections of human embryos (for example, see figure 23 on plate 3 of [50]). More recently, cells that are transcriptionally similar to the mouse AVE have also been identified in cultured human embryos [51]. Although with the formation of the bilaminar germ disc the human embryo acquires a flatter geometry compared to the mouse egg-cylinder, at the time of implantation and when the ‘DVE’ is likely to be induced, the human hypoblast and epiblast still show pronounced curvature (see figure 20 on plate 3 of [50]), making it conceivable that similar mechanical cues for DVE-induction based on curvature could be at play in both species at peri-implantation stages. Concurrently, supra-cellular structures similar to the actomyosin ring in the marginal zone of chick embryos [52] could also provide further directional mechanical cues to the hypoblast in a less geometry-dependant manner.

## Cell migration within epithelia

Cell migration is a fundamental process that underlies many developmental events, enabling either the dispersal of cells to new locations or the reorganisation of cells within a tissue. For tissues that are specialised to act as barriers, epithelia are remarkably dynamic at the cellular level, facilitating widespread rearrangement and movement of cells.

A striking example of migration of a sub-population of cells within the plane of an intact epithelial monolayer is the AVE, which collectively migrate from the distal-most tip of the egg-cylinder towards one side of the epiblast over the course of several hours [53]. From this position, AVE cells signal to the underlying epiblast through secreted inhibitors of Nodal and WNT pathways, preventing primitive-streak formation on their side of the embryo and thereby setting up the anterior-posterior axial asymmetry of the mammalian body plan [48,54].

Over the last 15 years, a combination of live imaging and genetic knockouts have revealed that AVE migration is an active process, controlled by coordinated differences in behaviour in sub-regions of the VE [53,55]. Knockouts of regulators of the actomyosin cytoskeleton block or cause aberrant migration of the AVE [56,57]. AVE cell migration is characterised behaviourally by an apicobasal divergence. Basally, cells send out highly dynamic cellular projections predominantly in the direction of migration [53,58] while apically, they retain intact tight-junctions and show WNT-PCP dependent actin localisation [55]. While the precise regulation of this migratory behaviour is unknown, it is clear that cells must coordinate apicobasal behaviour to enable dynamic remodelling of cell-cell contacts throughout migration, thereby retaining an intact epithelial monolayer. The fact that similar apicobasally polarised behaviours have also been observed in other epithelial contexts, for example in live imaging studies of mouse gut villi [59] and zebrafish optic cup rim cells [60], suggest that this type of migratory behaviour may be more widespread amongst epithelia than previously appreciated.

## Modulating epithelial integrity

In the blastocyst, the integrity of the epithelium itself can be a dynamic property. High blastocoel pressure leads to junctional leakages around cells undergoing mitosis, which, though momentarily disrupting epithelial integrity, helps to maintain the overall size of the blastocyst by restricting size oscillating within confined limits [22]. In the post-implantation embryo, despite the dynamic movement of cells within the VE, clonally-related cells remain in close proximity to each other with minimal neighbour exchange [61]. In contrast, within the pseudostratified epithelium of the pre-gastrulation epiblast, cells round up and move apically prior to mitosis. Epithelial integrity is maintained as neighbouring cells spread basally to fill the space, which necessitates the daughter cells to re-establish contact with the basal lamina leading to the gradual dispersal of clonal descendants [62]. Similar behaviours of cell mixing during mitosis of epithelial cells has also been described in other contexts, such as within the developing ureteric epithelium [63]. As an epithelium expands through mitosis of its component cells, mechanisms to facilitate the reintegration of the new daughter cells also need to be in place to ensure epithelial architecture and integrity are maintained. For example, within the epiblast, the ASPP2/PP1 complex is essential for this reintegration process by regulating cytoskeletal organisation at apical junctions [64]. ASPP2 plays an essential role in a variety of pseudostratified epithelia under mechanical stress, suggestive of a common molecular mechanism at play across these different tissues.

It is unclear why such dispersal of cells during division is seen in some epithelia such as the epiblast, but not others, such as the VE. It is possible that it only occurs in epithelia subject to particular levels of stress, or a particular geometry (e.g. only in pseudostratified, not simple, epithelia). In the case of the epiblast, the dispersal of cells could have a selective advantage as it would prevent the localised clonal accumulation of cells with deleterious mutations, which could otherwise impact on epithelial integrity if they are collectively elimination during the wave of cell competition that occurs prior to gastrulation [65,66]. It could also act as a mechanism through which the emergence of polarised heterogeneities within the epiblast are delayed, and in doing so, help fine-tune the timing of gastrulation. This could enable the VE, which does not show cell mixing, to develop coherent regionalised molecular differences well before the epiblast, facilitating the function of the former in patterning the latter [35].

## Disassembling and reassembling epithelia

During homeostatic maintenance of tissues, interconversion of epithelial and mesenchymal cells is rare and seen mostly only under pathological circumstances. However, during development, transitions between the two states occur frequently, and are used as a means through which cells originating in epithelia can move over larger distances and reassembled at new anatomical locations. This reduces the reliance on *de novo* pattering as the only means of generating new epithelia. In fact, the first migratory cell population in the embryo, the parietal endoderm (PE), is generated through delamination from the PrE, an epithelial tissue that arises from the ICM at ~E4.0. PE cells migrate along the inner surface of the TE, eventually contributing to extraembryonic structures of the foetus. Little is known about this first epithelial-to-mesenchymal transition (EMT) event, as the delamination of the PE coincides with the implantation of the embryo into the uterine wall, making it relatively intractable to experimentation.

However, this process is subsequently repeated multiple times during development, most notably at E6.25, during gastrulation. Here, cells in the pseudostratified epiblast undergo a fundamental cell fate choice, either delaminating to form the primitive streak through which cells move to give rise to the mesoderm and endoderm, or remain behind to commit to a default ectodermal differentiation pathway [33]. Major signalling pathways (including BMP, Nodal, WNT and FGF) and transcription factors (including EOMES, SNAIL and T) are required for primitive streak formation, but it is still unclear precisely how the initiation of primitive streak formation is controlled [54,67]. Prior to EMT, the epiblast basement membrane components (laminin and collagen IV) are degraded specifically on the posterior side due to the regionalised Nodal dependent expression of matrix metalloproteinases (MMP2 and MMP14) [68]. The perforation of the basement membrane is thought to make the epiblast here more prone to breaching. Individual cells within this region then downregulate E-cadherin and undergo a characteristic shape change, forming bottle-shaped cells, with their apical regions constricted as they move through the streak to form the mesoderm. From live imaging studies, it can be seen that the cells in the epiblast are relatively static prior to primitive streak formation [69,70], but have different packing organization from those in the anterior, with more rosettes forming in the posterior, through which a central cell can ingress [71]. Posterior epiblast cells also show a change in cell division behavior with the nuclei of cells no longer moving to the apical-most region to divide, but rather dividing medially (i.e., away from the apical region) [71]. Together these new insights suggest that biomechanical changes occur across the epiblast, involving basement membrane remodeling, cell packing and division events in concert with the molecular control of EMT to down-regulate epithelial characteristics.

The mesenchymal endoderm progenitors, generated at gastrulation through EMT, later re-epithelialise through mesenchymal-to-epithelial transition (MET) to give rise to the definite endoderm (DE) which intercalates with the existing VE epithelium, collectively to form the gut tube [37]. Recently it has been suggested that when emerging from the epiblast, endodermal precursors undergo a *partial* EMT [72], which might facilitate their rapid subsequent re-epithelialisation when forming the DE. During such transitions, epithelia loosen their junctional interactions and transiently behave as individual, yet polarised mesenchymal-like intermediates. This supports the notion that epithelial and mesenchymal states represent opposite extremes of a morphological continuum that cells occupy, related to their behaviour and molecular state [73,74].

## Conclusion and perspectives

Epithelia are dynamic structures that must constantly reconcile two opposing imperatives; structural integrity and behavioural flexibility. Understanding how epithelial cells achieve this is important for understanding embryonic morphogenesis during development, and also in providing a route for identifying the molecular machinery relevant to pathological transformations. Great strides have been made in unravelling the molecular and cellular biology of embryonic epithelial behaviour. In the future, advances in imaging, biomechanical and biochemical perturbation and computational modelling approaches may help us connect the molecular to the biophysical modulation of epithelial dynamics.
